# A Curious Case of Medium-Vessel Vasculitis

**DOI:** 10.7759/cureus.69111

**Published:** 2024-09-10

**Authors:** Nisa N Ilsin, Haleigh D Stafford, Anna Catinis, Theodore Rosen

**Affiliations:** 1 School of Medicine, Baylor College of Medicine, Houston, USA; 2 Department of Dermatology, Baylor College of Medicine, Houston, USA

**Keywords:** cacti, case report, interleukin-17, skin ulcer, spider bite, treatment delay, vasculitis

## Abstract

Vasculitis, or the inflammation of vessels due to primary or secondary causes, may arise from numerous etiologies, often leading to diagnostic uncertainty. Delayed treatment due to diagnostic or etiologic uncertainty presents a significant clinical risk, with consequences including organ failure and mortality. We describe a case of a 58-year-old male with a history including ankylosing spondylitis who presented with painful ulcers involving the bilateral lower extremities following a trip to the southern Texas border. Histopathology revealed medium-vessel vasculitis; however, the search for a likely etiology in the setting of a unique combination of potential vasculitis precipitants, including glochid inoculation, a spider bite, prior IL-17 inhibitor use, and inflammatory bowel disease, contributed to treatment delay and disease progression. Although the patient was ultimately successfully treated with systemic corticosteroids, this case highlights the importance of initiating prompt therapy once vasculitis is recognized to prevent disease progression, even if lacking an identified etiology.

## Introduction

Vasculitis describes a broad group of primary and secondary disorders characterized by the inflammation and necrosis of small, medium, or large blood vessels [1,2]. Vasculitides are more common in the adult population, with an estimated annual incidence of 20 to 40 million individuals affected by primary vasculitis in Europe and the United States alone [3,4]. Medium-vessel vasculitis primarily involves the main visceral blood vessels of the arterial and venous systems. It has a vast array of etiologies, ranging from direct vessel pathology to infectious and medication-induced vascular destruction [4,5]. For example, medium-vessel vasculitides polyarteritis nodosa (PAN) and Kawasaki disease are precipitated by viral etiologies, such as the association of hepatitis B virus with PAN [1,4]. The clinical presentation of medium-vessel vasculitis is similarly heterogeneous; however, common features include dermal manifestations mostly observed on the lower extremities, such as necrotic papules or ulcerations, and systemic manifestations, such as fever and weight loss [4]. Due to this heterogeneity, there is no single reliable diagnostic algorithm for vasculitides [6]. Diagnosis predominantly depends on clinical and histologic evaluation and correlation, with biopsy being key for diagnostic confirmation. However, the subsequent workup involving laboratory analysis and imaging to determine the underlying etiology is often considered equally important [4].

Initial treatment of vasculitis typically involves systemic corticosteroids and other immunosuppressive agents, such as methotrexate, rituximab, azathioprine, or cyclophosphamide, and the time to treatment initiation remains a critical factor in patient prognosis [1,3,4]. Delayed treatment due to diagnostic or etiologic uncertainty presents a significant clinical risk, with consequences including organ failure and mortality [4,7]. Herein, we describe the case of a 58-year-old male who presented with complex dermal and systemic manifestations of medium-vessel vasculitis of an undetermined cause, leading to treatment delay. This case emphasizes the importance of early treatment for vasculitis in the presence of high clinical suspicion to optimize patient outcomes.

## Case presentation

**Figure 1:**
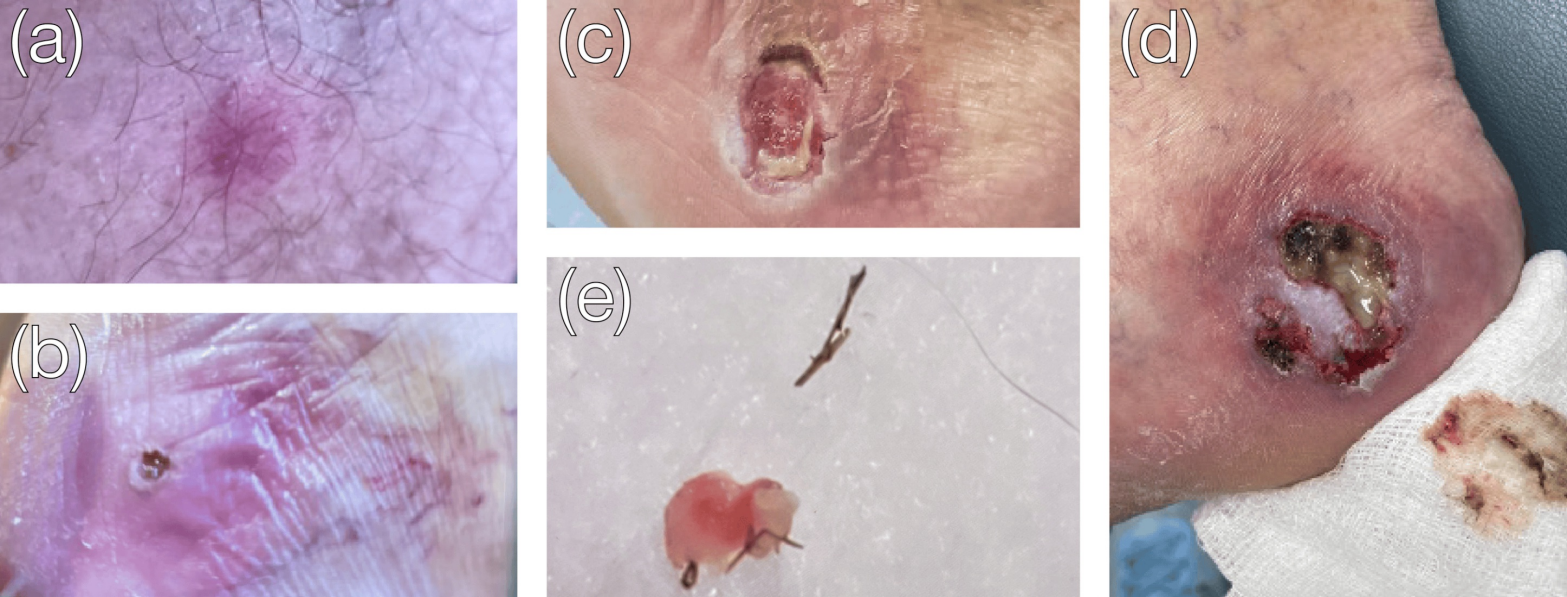
Progression of the patient's right medial heel wound. The lesion initially began as a small, erythematous papule, similar to this later-developing lesion on the upper right leg (a). It quickly developed a central black eschar (b) before ulcerating (c), enlarging, and becoming necrotic (d). A cactus spine was removed from the right heel wound (e).

A 58-year-old male with a past medical history of diabetes mellitus, ankylosing spondylitis, and remote tobacco use was evaluated for three months of a progressive, extremely tender right medial heel wound and newly developing ulcerated subcutaneous nodules spreading superiorly up his lower right leg. The patient first noticed these lesions shortly after a hunting trip to the southern Texas border (between Laredo and Hebbronville, Texas). During this trip, he traversed a cactus forest where he was stuck by innumerable cacti spines. He also reported finding a brown spider in his right shoe a few days later. Shortly after, he felt pain in his right medial heel. Three days after symptom onset, he noticed a painful erythematous papule that developed a central eschar and progressed into an ulcerated plaque with inflammatory borders, prompting his initial presentation to an emergency department (Figure 1). He was discharged with a brief course of amoxicillin-clavulanate followed by clindamycin and was advised to stop taking ixekizumab, the biologic therapy prescribed for ankylosing spondylitis, which was started just prior to his hunting trip. The wound persisted for months despite several additional courses of antibiotics and routine wound care, and his condition progressed to involve several similar lesions extending up his right leg, each beginning as an erythematous papule that developed a central black eschar before ulcerating. During this time, he also experienced new-onset non-bloody diarrhea and an unintentional 20-pound weight loss, prompting a hospital admission, which led to consultation with dermatology.

Upon our initial evaluation, the patient was tachycardic, febrile, and with severe, refractory pain to his right lower extremity, impacting his ability to ambulate. The dermatologic evaluation confirmed the presence of an ulcerated plaque with central yellow granulation tissue and surrounding erythema to the right medial heel. Newer erythematous papulonodules with central black eschars were also noted along the right medial upper leg. Laboratory studies were notable for leukocytosis to 15.0 x 10^9^/L and an elevated erythrocyte sedimentation rate and C-reactive protein. At this time, the differential diagnosis primarily included ecthyma, indolent fungal or atypical mycobacterial infection in the setting of possible inoculation from the cacti spines or presumed spider bite, and vasculitis. Punch biopsies were obtained from two separate lesions for histology and routine cultures, and the patient was discharged with antibiotics per the recommendations of the consulting infectious disease team. Initial histopathology revealed ulceration and fibrinoid dermal necrosis with vasculitic changes and mixed inflammatory infiltrates in both lesions with negative stains and tissue cultures for both fungi and mycobacteria.

**Figure 2:**
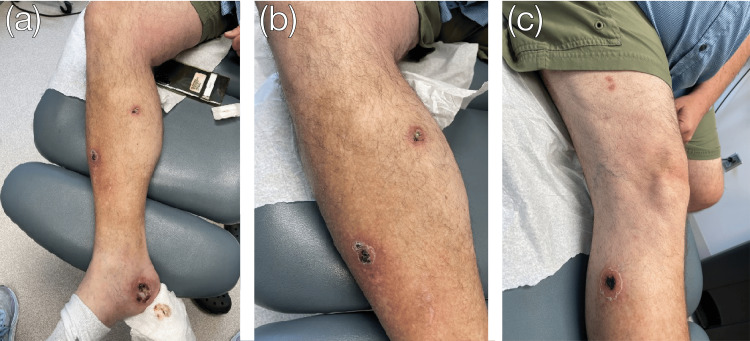
Sporotrichoid-appearing dissemination of ulcerated lesions. Wound progression began with the initial lesion on the right medial heel (a) and progressed superiorly to the right calf (b) and thigh (c).

**Figure 3:**
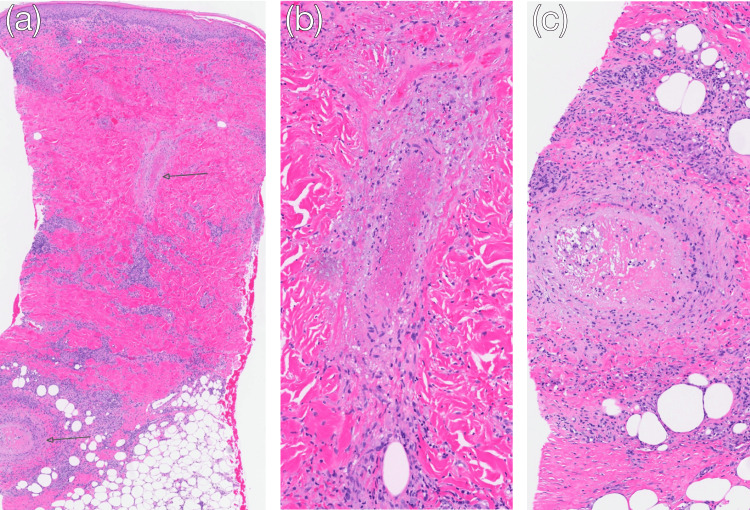
Histopathology of right lower extremity lesions. (a) Low-power magnification revealing medium- and large-vessel vasculitis (hematoxylin and eosin (H&E), magnification ×20); (b) high-power magnification showing a medium-sized vessel in the dermis with vasculitis, cellular debris, and fibrinoid vessel necrosis (H&E, magnification ×100); (c) high-power magnification showing large-vessel vasculitis in subcutaneous tissue, with fibrinoid necrosis and some focal granulomatous inflammation around (H&E, magnification ×100).

The patient was followed in the outpatient setting, and his clinical status continued to decline with worsening pain and new lesions arising in a seemingly sporotrichoid pattern up the right lower extremity (Figure 2). This prompted repeat biopsies for histology and cultures, given increasing concern for atypical infection. Histology revealed a subcutaneous lymphocytic and histiocytic infiltrate with medium-vessel vasculitis and unremarkable microbiological stains (Figure 3). Tissue cultures were again negative, and an extensive rheumatologic workup, including testing for known markers of autoimmune disorders and vasculitides such as antinuclear, anti-myeloperoxidase, and antineutrophil cytoplasmic antibodies, was also unrevealing. The patient was ultimately re-admitted for pain control and further workup of his progressing cutaneous lesions, now involving the bilateral lower extremities. Infectious disease signed off, given the patient's lack of response to numerous courses of antibiotics and repeatedly negative tissue cultures. Our team recommended corticosteroid initiation for the treatment of vasculitis; however, therapy was withheld amid the continuing search for an etiology, and additional biopsies were requested for direct immunofluorescence (DIF). Repeat biopsies confirmed the diagnosis of medium-vessel vasculitis, and DIF was unremarkable.

**Figure 4:**
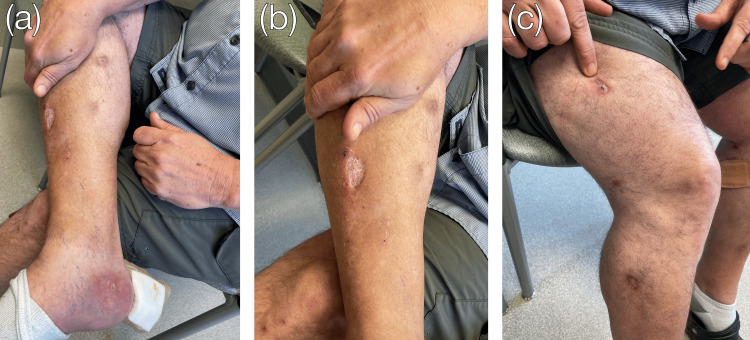
Healed lesions following corticosteroid therapy. Marked healing of the right medial heel wound (a) and the superiorly spreading right calf (b) and thigh (c) lesions were seen after treatment with IV methylprednisolone and a subsequent oral prednisone taper.

The patient's hospital course was complicated by new-onset large-volume hematochezia, prompting colonoscopy with intestinal biopsies, which revealed pancolitis and granulomatous inflammation, respectively. The patient was diagnosed with Crohn's colitis, thought to have been previously masked by years of immunosuppressive therapies for his known ankylosing spondylitis. The patient was subsequently started on a five-day course of 20 mg IV methylprednisolone every eight hours for both Crohn's colitis and medium-vessel vasculitis. The administration of corticosteroids led to rapid symptomatic improvement, and he was discharged with oral prednisone 40 mg daily with a 5 mg weekly taper and planned to transition to upadacitinib for long-term management of his spondylitis. On follow-up, the patient reported a dramatic recovery, with marked healing of cutaneous lesions, significant improvement in pain, and a restored ability to ambulate (Figure 4). However, while upadacitinib successfully controlled his Crohn's colitis, his lower extremity lesions flared within a week of finishing the prednisone taper, prompting a prolonged regimen of oral steroids.

## Discussion

Vasculitis may be of primary or secondary etiology, triggered by medications or infections, for example, leading many cases to be diagnosed and treated without ever identifying a definitive cause [4,5]. The diagnosis of vasculitis is generally based on the size of affected vessels, histopathologic features, and additional data from laboratory analysis and imaging [4]. While a potential etiology or trigger may help focus one's therapeutic plan, a more detailed diagnostic approach should not deter treatment initiation once a specific type of vasculitis is suspected. Nevertheless, the vast array of potential etiologies may present a barrier to timely management, as was the case for our patient. Of the differentials considered, secondary immunologic response to cactus spines, presumed spider bite, ixekizumab IL-17 blockade, and underlying inflammatory bowel disease (IBD) were suspected possibilities.

While there are no known cases of cactus spine-induced vasculitis, envenomation from the brown recluse spider, endemic to the southern United States, is a well-documented precipitant of vasculitis [8-10]. Another spider endemic to the region is the black widow spider, the venom of which has similarly been shown to prompt vasculitis [11]. Although an intriguing potential etiology, spider venom could not be eliminated nor confirmed as the cause of vasculitis in this case.

Ixekizumab-induced vasculitis was also considered due to its initiation a few weeks prior to the patient's symptom onset, especially with known reports of drug-induced vasculitis from IL-17 inhibitors [12]. Perivascular IgA deposition is associated with vasculitis from IL-17 blockade, prompting evaluation with DIF late in the patient's hospital course [13]. In our case, the absence of immunoglobulin deposition essentially excluded the possibility of IL-17 inhibitor-related vasculitis. Finally, his newly uncovered Crohn's colitis presents a fourth potential trigger, as vasculitis has been reported to occur in the setting of IBD [14]. However, we deemed this an unlikely etiology, considering his cutaneous lesions flared once tapered off systemic steroids despite control of his IBD with upadacitinib, necessitating a prolonged steroid course.

Given the plethora of possible vasculitis triggers, in addition to the initial suspicion of indolent infection, treatment was delayed in our patient's case, which allowed for disease progression while an extensive and largely unrevealing workup was performed. In addition to the unique nature of numerous interesting potential vasculitic triggers in a single patient, the clinical significance of this case lies in the message that vasculitis should be treated promptly when recognized, even without an established underlying cause. In one study, delayed treatment of vasculitis was associated with 82% of participants reporting delays that negatively impacted their health, resulting in disability and/or worsening health outcomes [7]. Diagnostic delays of vasculitis are associated with an increase in morbidity and mortality through pulmonary manifestations such as alveolar hemorrhage, gastrointestinal bleeding, septic shock, and/or organ failure [15]. With such critical consequences of treatment delay, it is pivotal to ensure that the search for an exact etiology does not impede the rapid treatment of vasculitides. While long-term management of vasculitis may vary depending on the underlying cause, corticosteroids with subsequent steroid titration remain the first-line choice, and, if necessary, immunosuppressive agents such as methotrexate or rituximab can be added to target a specific etiology of vasculitis [3].

## Conclusions

Vasculitis can have devastating consequences if not rapidly identified and treated, with progressively worsening manifestations at the affected sites and a risk of organ failure. Due to the many possible causes of vasculitis, diagnostic delay in the presence of etiologic uncertainty proves to be an ongoing barrier to successful treatment, as illustrated in this case in which the investigation of multiple complex exposures led to treatment late in the clinical trajectory. Despite challenges in identifying a vasculitic trigger, physicians should prioritize the early treatment of vasculitis in the context of an unspecified precipitant to ensure optimal health outcomes.
